# Effects of Dietary Fatty Acids on Bovine Oocyte Competence and Granulosa Cells

**DOI:** 10.3389/fendo.2020.00087

**Published:** 2020-02-25

**Authors:** Arpna Sharma, Vijay Simha Baddela, Volker Roettgen, Andreas Vernunft, Torsten Viergutz, Dirk Dannenberger, Harald M. Hammon, Jennifer Schoen, Jens Vanselow

**Affiliations:** Reproductive Biology Unit, Leibniz Institute for Farm Animal Biology (FBN), Dummerstorf, Germany

**Keywords:** alpha-linolenic acid, conjugated linoleic acid, estradiol, granulosa cells, gene expression

## Abstract

Here we assessed the effects of dietary essential fatty acids on the developmental competence of oocytes in cows and on the functionality of follicular granulosa cells (GC). Lactating German Holstein cows were supplemented from week 9 ante partum (ap) until week 8 post-partum (pp) in four dietary groups designed as (i) control (CTRL: coconut oil), (ii) essential fatty acid (EFA: linseed and safflower oil), (iii) conjugated linoleic acid (CLA: Lutalin®), and (iv) EFA+CLA (mixture of linseed oil, safflower oil and Lutalin®). EFA, CLA or EFA+CLA supplementation did not improve *in vitro* embryo production. However, higher proportions of α-linolenic acid (ALA) and *cis-9, trans-11* CLA were observed in the follicular fluid suggesting the exposure of GC to relatively high levels of ALA and *cis-9, trans-11* CLA. Consequently, we tested different concentrations of ALA and *cis-9, trans-11* CLA in a bovine GC culture model for their effects on steroid production, marker gene expression and viability. Both fatty acids upregulated *CD36* and downregulated the expression of *FOXL2*, while ALA significantly increased *SOX 9* transcript levels. Both ALA and *cis-9, trans-11* CLA reduced the *CCND2* expression and *cis-9, trans-11* CLA induced apoptosis. ALA and *cis-9, trans-11* CLA significantly down-regulated the expression of *STAR, CYP19A1, FSHR, LHCGR* and decreased the 17β-Estradiol (E2) and progesterone (P4) production. In conclusion, dietary lipids did not improve *in vitro* embryo production, while ALA and *cis-9, trans-11* CLA affected the morphology and functionality of GC. This could suggestively lead to compromised follicle development and ovarian cyclicity in dairy cows.

## Introduction

Dietary supplements can improve the reproductive outcome in cows by increasing the energy intake thus reducing the extent of negative energy balance (NEB) experienced postpartum (1). Polyunsaturated fatty acids (PUFA) enriched diets in cattle are known to alleviate NEB during early lactation (2). PUFA at the cellular level maintain several functions such as membrane stability, regulation of transcription factors, cell proliferation and differentiation (3). Among PUFA, essential fatty acids such as linoleic acid (LA) and α-linolenic acid (ALA) are not synthesized in cows, human and pigs and must be supplied through the diet (4). *In vivo*, the nutritional effects of PUFA are well-known to affect the oocyte metabolism and early development in livestock species (5). The fatty acid (FA) profile of the follicular fluid (FF) is well-correlated to the type of dietary FA surrounding the oocytes as dietary fats can alter the FA composition in cumulus and granulosa cells (GC) thus influencing oocyte quality (6). Ewes fed with PUFA diets showed higher proportions of LA and docosahexaenoic acid (DHA) in FF and cumulus cells with lower proportions in oocytes (7). The size of dominant follicles is reported to increase in cows fed with PUFA diets as compared to cows fed with monounsaturated fatty acids (MUFA) (8). Increased follicle size is supposed to improve both oocyte quality and corpus luteum (CL) function in cows (9). Larger ovulatory follicles with lower rates of pregnancy losses were found in cows fed with flaxseed (9.8%) compared to those fed with sunflower seed (27.3%) (10). Among other dietary fats, conjugated linoleic acid (CLA) isomers (*cis*-*9, trans-11* and *trans-10, cis-12*) comprise a group of PUFA derived from LA during incomplete biohydrogenation by the ruminal flora in cattle and sheep (11). CLA increases the plasma concentration of insulin-like growth factor-1 (IGF-1) in cows, thus promoting conception rates (12). CLA supplements decrease the milk fat excretion during the early lactation period saving energy in order to combat the physiological NEB (13). As CLA at physiological concentrations has been found to affect nuclear maturation of cumulus oocyte complexes (COCs) and similarly, LA could also negatively affect both oocyte and embryo development due to altered glutathione peroxidase and superoxide dismutase mRNA expression (14). Negligible effects of ALA and LA diets on cleavage and embryo development from oocytes collected *via* ovum pick up (OPU) have been reported (15). Cows fed with omega-3 (ω-3) enriched diet failed to enhance the ovulation even upon ovarian stimulation (16). Dietary effects of essential fatty acids particularly ALA and CLA or their mixture on oocyte developmental competence as well as their effects on GC functionality still remain to be elucidated in detail. Thus, the primary objective of the study was to determine the effect of essential fatty acids especially of ALA and CLA by abomasal supplementation in lactating cows to assess the effects on *in vitro* preimplantation embryo development. We also analyzed the FA compositions of FF in the different dietary groups and elucidated the effects of increased concentrations of ALA and *cis-9, trans-11*CLA on the morphology, hormone production, viability, and gene expression of cultured bovine GC.

## Materials and Methods

### Animals and Dietary Supplementation

Handling of animals and the experimental design were approved by the federal state of Mecklenburg Western-Pommerania, Germany (LALLF M-V TSD 7221.3-1-038/15). Forty German Holstein-Friesian cows at 18th week of gestation in their 2nd lactation were kept in a free-stall barn at the Leibniz Institute for Farm Animal Biology (FBN), Dummerstorf, Germany. Cows included in the study were surgically fitted with rumen fistulas and abomasal tubes to bypass rumen derived biohydrogenation of supplemented FA (17). The cows were studied in 5 blocks each consisting of 8 cows (2 cows per supplementation group and per block) from week 9 ante partum (ap) up to week 8 post-partum (pp) in their 3rd lactation. The ingredients and the composition of the diet, separately for lactation and dry-off diet are shown in Supplementary Table 1. FA composition of all four oil supplements is shown in Supplementary Table 2. Cows were supplemented daily from week 9 ap until week 8 pp either with 76 g/d coconut oil (CTRL *n* = 9), 78 g/d linseed and 4 g/d safflower oil (EFA *n* = 9) providing a ω-6/ ω-3 FA ratio of 1:3, Lutalin® (CLA *n* = 10, 10 g/d of *cis-9, trans-11*, and *cis-10, trans-12* CLA isomer, respectively) or a mixture of linseed and safflower oil plus Lutalin® (EFA + CLA *n* = 10). Following last dietary supplementation, cows in wk 9 pp were slaughtered for oocyte collection by the slicing method. In between, due to abortions in week 6 ap, 4 cows had to be removed from the study though two of these cows were replaced. Thus, altogether 38 cows were examined throughout the experimental duration.

### FA Analysis by Gas Chromatography

FF from large follicles (10–24 mm) was aspirated from ovaries of the experimental cows after slaughter. Approximately 500 μL of FF was dropwise added to 8 mL chloroform/methanol (2:1, v/v) at room temperature. The fatty acid C19:0 used as an internal standard. The procedure for sample preparation was performed as described earlier (18). The fatty acid analysis of the FF was executed using capillary gas chromatography with a CP-Sil 88 CB column (100 m × 0.25 mm, Chrompack-Varian, Lake Forest, CA, USA) which was installed in a PerkinElmer gas chromatograph Autosys XL with a flame ionization detector (PerkinElmer Instruments, Shelton, CT, USA). The complete gas chromatography conditions were as described earlier (19). Hydrogen at a flow rate of 1 mL/min was used as the carrier gas while the split ratio was 1:20 with the injector and detector set to 260 and 280°C, respectively.

### *In vitro* Production (IVP) of Embryos

The IVP protocol was executed using the IVF Bioscience media suite formulated for bovine embryo production according to the manufacturer's instructions (IVF Bioscience, Falmouth, United Kingdom, catalog # 61002, 61004, 61003, 61010, 61001, and 62000) (20). Briefly, 6–10 COCs obtained from cows of different diet groups after slaughter were matured in BO-IVM media and incubated for 24 h (38.5°C, 6% CO_2_). For IVF, 2 × 10^6^ sperms/ml from proven fertile Holstein Friesian bull were added to BO-IVF media containing matured oocytes and incubated for 18–22 h (38.8°C, 6% CO_2_, 21% O_2_). Putative zygotes were cultured in BO-IVC media and overlaid by BO-Oil and incubated at 38.8°C, 6% CO_2_, 6% O_2_, 88% N_2_. Embryo cleavage was examined 48 h post IVF and subsequent embryo development was evaluated on day 8 post IVF. To count total cell number (TCN), embryos were fixed in 4% (v/v) paraformaldehyde (Sigma 252549) and stored at 4°C overnight. Embryos were mounted on glass slides and stained with Hoechst 33258 (Sigma B1155). Images were captured by confocal laser scanning microscope LSM 800 assembled with ZEN software (Carl Zeiss, Oberkochen, Germany).

### Primary GC Culture

Primary GC culture and bovine serum albumin (BSA)-FA conjugate preparations were done as described earlier (21). For testing the effects of actual follicular concentration of ALA (Sigma-Aldrich,L2376) and *cis-9, trans-11*CLA (Sigma-Aldrich,16413) the media were replaced every 48 h with supplemented α-MEM media containing different concentrations of ALA (20, 40, and 80 μM) or *cis-9, trans-11* CLA (15, 30, and 60 μM) as BSA conjugates or non-conjugated BSA as vehicle control (Sigma-Aldrich A7030). All control wells received the same volume of BSA as the test groups with highest fatty acid concentration. The conditioned media collected on the 8th day of culture were stored at −20°C for steroid hormone estimation, while the remaining cells were lysed for RNA isolation.

### Steroid Hormone Analysis

Concentration of E2 and P4 of GC conditioned media were determined by competitive 3H–radioimmunoassay (RIA) with rabbit-raised antibodies purified by affinity chromatography as performed earlier (22). Radioactivity was measured in a liquid scintillation counter (LSC) with an integrated RIA-calculation programme (TriCarb 2900 TR; PerkinElmer).

### Flow Cytometry

On day 8 of GC culture, cells were thoroughly washed twice with phosphate buffered saline (PBS) and then trypsinized using 250 μl TryplE solution (Thermo Fischer, USA) at 37°C for 20 min. Post trypsinization, all detached cells were centrifuged and resuspended in 1 ml MEM and analyzed for viability and apoptosis using an Annexin-V FITC/PI kit (Miltenyi biotec, Germany). Cells were centrifuged and pellets were re-suspended in 100 μl of binding buffer to which 10 μl of Annexin V reagent was added and kept for incubation in the dark for 15 min. followed by washing and resuspension in 500 μl binding buffer. Next, 5 μl of PI (Propidium iodide, 500 μg/ml) was added to the cells with gently mixing just prior to flow cytometric analysis. The fluorescence signal was quantified from single cells (10,000 counts) by a flow cytometer (Gallios, Beckman-Coulter, Germany) and the data obtained was analyzed using the Kaluza-software (Beckman-Coulter, Germany).

### RNA Isolation, cDNA Synthesis, and Real Time-Quantitative PCR (RT-qPCR)

RNA isolation was performed using the innuPREP RNA Mini Kit (Analytik Jena, Germany) according to the manufacturer's protocol and quantified with NanoDrop1000 Spectrophotometer (Thermo Scientific, Bonn, Germany). Later, cDNA was synthesized using the SensiFAST cDNASynthesis Kit (Bioline, Luckenwalde, Germany) from 200 ng RNA as done previously (23). The RT-qPCR was executed for gene expression analysis, using SensiFAST SYBR No-ROX (Bioline) with gene-specific primers (Supplementary Table 3) in a Light Cycler 96 instrument (Roche, Mannheim, Germany) (24).

### Statistical Analysis

IVF data were analyzed by one-way ANOVA (Holm-Sidak method, all pair wise multiple comparison procedure). The mean proportions of follicular fatty acids in supplemented diet groups was compared by one way ANOVA (Holm-Sidak method) or ANOVA on ranks (Dunn's Method) for pairwise multiple comparisons. The RIA, RT-qPCR gene expression and flow cytometry data (at least three biological replicates) were analyzed by one-way repeated measure ANOVA (all pair wise multiple comparison or multiple comparison vs. control procedures with Holm-Sidak or Dunnett test where applicable) or ANOVA on ranks when normality test failed using SigmaPlot 11.0. Significant changes were recognized if *P* < 0.05.

## Results

### FA Analysis of Dietary Lipid Supplements in Follicular Fluid

The proportions of different FA were analyzed in the FF of all four dietary groups (Table 1). The percentage of ALA was significantly (*P* < 0.05) higher in the EFA and EFA + CLA diet groups with 10.19 ± 2.36 % and 12.56 ± 2.02%, respectively, as compared to the CTRL (2.81 ± 0.46 %) and CLA diet groups (2.44 ± 0.45 %). While *cis-9, trans-11* CLA isomer was significantly higher (*P* < 0.05) in the CLA and EFA+CLA diet groups with 0.4 ± 0.12% and 0.29 ± 0.03%, as compared to the CTRL and EFA diet groups (0.15 ± 0.15% and 0.07 ± 0.03%). Further, due to higher proportions of ALA and *cis-*9, *trans-*11 CLA in FF, we speculate that GC are exposed to relatively high levels of ALA and *cis-*9, *trans-*11 CLA which might affect the functionality of GC residing within. Consequently, we tested different concentrations of both ALA and *cis-9, trans-11* CLA in our GC culture model.

**Table 1:** Fatty acid profile of follicular fluid analyzed by Gas chromatography.

**Fatty acids**	**CTRL**	**CLA**	**EFA**	**EFA+CLA**
**Saturated fatty acids (SFA)**				
C10:0	0.24 ± 0.1	0.23 ± 0.21	0.28 ± 0.22	0.26 ± 0.23
C11:0	0.01 ± 0.005	0.009 ± 0.003	0.02 ± 0.03	0.01 ± 0.006
C12:0	0.33 ± 0.11	0.27 ± 0.07	0.31 ± 0.11	0.26 ± 0.1
C13:0	0.02 ± 0.01	0.03 ± 0.01	0.04 ± 0.01	0.03 ± 0.01
C14:0	1.1 ± 0.19^**ab**^	1.26 ± 0.19^**a**^	1 ± 0.16^**b**^	1.14 ± 0.3^**ab**^
C15:0	0.63 ± 0.12	0.73 ± 0.12	0.72 ± 0.16	0.73 ± 0.18
C16:0	10.95 ± 0.66	11.38 ± 1.09	12.89 ± 2.19	11.42 ± 2.1
C17:0	0.86 ± 0.24	0.84 ± 0.17	0.87 ± 0.23	0.93 ± 0.23
C18:0	11.21 ± 1.1	11.62 ± 1.08	10.66 ± 1.03	10.51 ± 1.2
C20:0	0.18 ± 0.07	0.22 ± 0.05	0.25 ± 0.05	0.23 ± 0.08
C21:0	0.13 ± 0.09^**a**^	0.23 ± 0.11^**b**^	0.14 ± 0.03^**ab**^	0.26 ± 0.08^**ab**^
C22:0	0.45 ± 0.14	0.56 ± 0.12	0.59 ± 0.16	0.58 ± 0.21
C23:0	0.21 ± 0.06	0.33 ± 0.16	0.73 ± 0.91	0.33 ± 0.23
C24:0	0.84 ± 0.31	0.98 ± 0.27	1.09 ± 0.26	1.01 ± 0.3
**Sum SFA**	27.22 ± 1.15	28.8 ± 1.68	29.65 ± 3.62	27.75 ± 3.98
**Monounsaturated fatty acids (MUFA)**				
C14:1*cis*-9	0.16 ± 0.27^**a**^	0.02 ± 0.01^**ab**^	0.02 ± 0.01^**b**^	0.07 ± 0.16^**b**^
C16:1*cis*-9	1.66 ± 0.3^**a**^	1.38 ± 0.27^**a**^	1.29 ± 0.14^**a**^	0.93 ± 0.09^**b**^
C17:1*cis*-9	0.43 ± 0.05^**a**^	0.32 ± 0.27^**ab**^	0.17 ± 0.1^**b**^	0.14 ± 0.17^**b**^
C18:1*cis*-9	8.78 ± 1.43^**a**^	7.67 ± 1.63^**a**^	8.2 ± 3.46^**a**^	5.15 ± 1.8^**b**^
C18:1*cis*-11	0.9 ± 0.16^**a**^	0.78 ± 0.17^**ab**^	0.89 ± 0.36^**ab**^	0.6 ± 0.11^**b**^
C18:1*trans-*9	0.11 ± 0.05^**a**^	0.08 ± 0.04^**b**^	0.06 ± 0.01^**b**^	0.07 ± 0.01^**ab**^
C18:1*trans-*11	0.5 ± 0.37	0.36 ± 0.08	0.32 ± 0.07	0.44 ± 0.2
C20:1*cis*-11	0.04 ± 0.01	0.04 ± 0.01	0.07 ± 0.07	0.03 ± 0.01
**Sum MUFA**	12.75 ± 1.5^a^	10.88 ± 2.06^a^	11.18 ± 3.92^a^	7.51 ± 1.92^b^
**Polyunsaturated fatty acids (PUFA)**				
C18:2*n*-6	49.06 ± 2.32^**ac**^	49.56 ± 2.88^**a**^	41.93 ± 5.74^**b**^	44.83 ± 4.21^**bc**^
**18:2*****cis*****-9**, ***trans*****-11 (CLA)**	0.15 ± 0.15^ac^	0.4 ± 0.12^b^	0.07 ± 0.03^a^	0.29 ± 0.03^bc^
**C18:3*****n*****-3**	2.81 ± 0.46^a^	2.44 ± 0.45^a^	10.19 ± 2.36^b^	12.56 ± 2.02^b^
C18:3*n*-6	0.6 ± 0.22^**a**^	0.41 ± 0.09^**ac**^	0.3 ± 0.12^**bc**^	0.12 ± 0.01^**b**^
C18:4*n*-3	0.04 ± 0.02^**ab**^	0.04 ± 0.02^**a**^	0.07 ± 0.02^**b**^	0.03 ± 0.01^**a**^
C20:2*n*-6	0.18 ± 0.13^**a**^	0.36 ± 0.1^**b**^	0.15 ± 0.08^**a**^	0.19 ± 0.04^**a**^
C20:3*n-*6	1.96 ± 0.53^**a**^	1.7 ± 0.5^**a**^	1.19 ± 0.28^**b**^	0.7 ± 0.11^**c**^
C20:4*n*-6	2.25 ± 1.2^**ab**^	2.7 ± 0.85^b^	1.68 ± 0.21^**c**^	1.9 ± 0.29^a**c**^
C20:5*n*-3	0.53 ± 0.13^**a**^	0.56 ± 0.14^**a**^	1.43 ± 0.29^**b**^	1.44 ± 0.29^**b**^
C22:4*n*-6	0.37 ± 0.18^**a**^	0.42 ± 0.15^**a**^	0.14 ± 0.05^**b**^	0.15 ± 0.07^**b**^
C22:5*n*-3	1.22 ± 0.23^**a**^	1.19 ± 0.39^**a**^	1.67 ± 0.47^**b**^	1.98 ± 0.48^**b**^
C22:6*n*-3	0.06 ± 0.02^**a**^	0.12 ± 0.02^**ac**^	0.12 ± 0.03^**bc**^	0.23 ± 0.09^**b**^
**Sum PUFA**	59.86 ± 1.95	59.9 ± 3.15	59.08 ± 6.6	64.43 ± 4.83
**Sum** ***n*****-3 PUFA**	5.29 ± 1.33^a^	4.63 ± 1.32^a^	13.58 ± 2.73^b^	16.43 ± 2.13^b^
**Sum** ***n*****-6 PUFA**	54.49 ± 2.57^a^	55.2 ± 2.74^a^	45.45 ± 5.81^b^	47.95 ± 4.16^b^

*Values are % of total fatty acids, expressed as means ± SD. The one way ANOVA (Holm-Sidak method) or ANOVA on ranks (Dunn's Method) for pairwise multiple comparisons were used for comparing means. Values with different superscripts on the same row are significantly different (P < 0.05). Diet groups are CTRL: coconut oil; EFA: linseed oil + safflower oil; CLA: Lutalin® and EFA + CLA: linseed oil + safflower oil + Lutalin®*.

### *In vitro* Embryo Development

**Figure 1:**
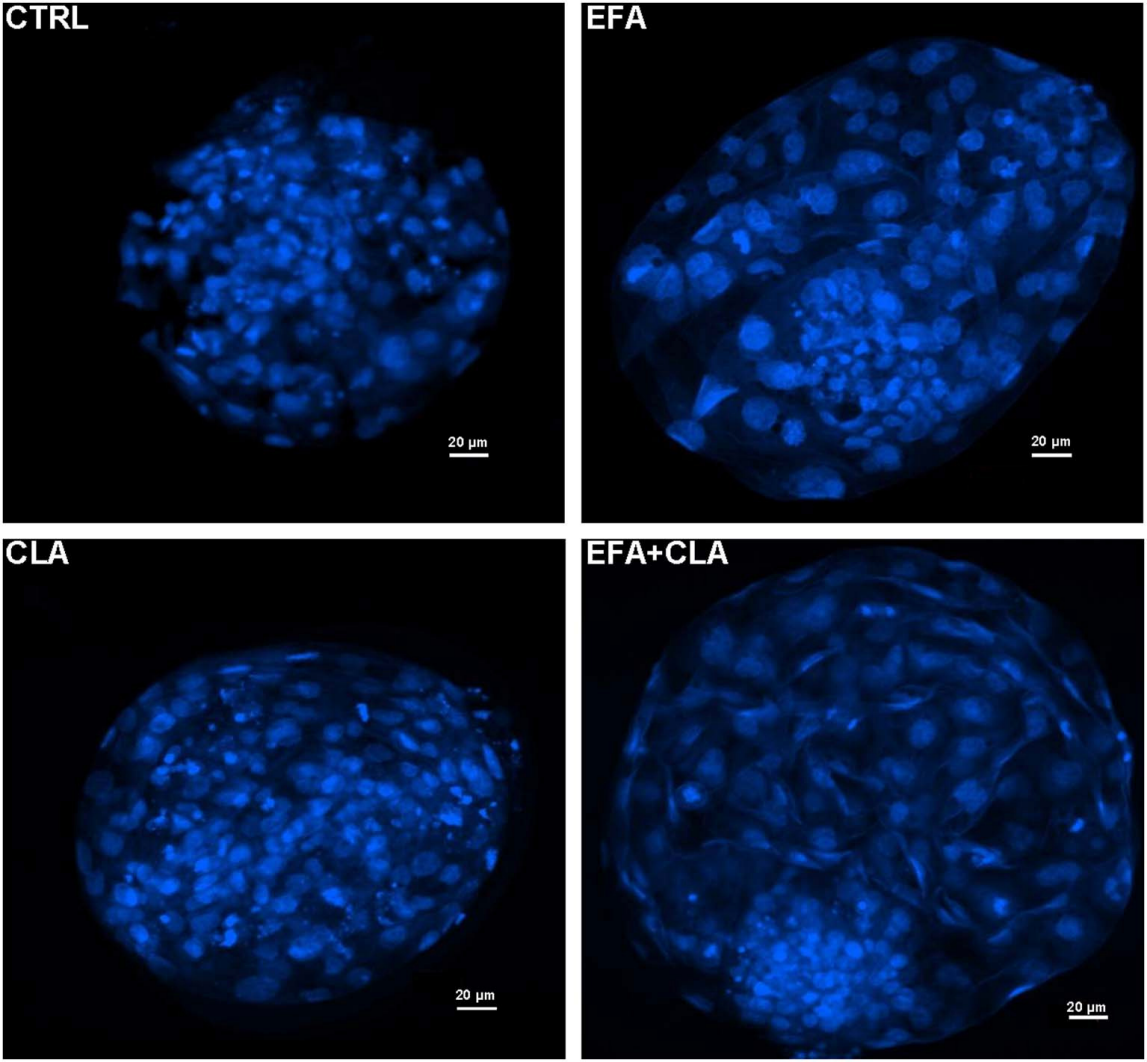
Fluorescence images of Hoechst 33258 stained blastocyst nuclei generated from different dietary groups. CTRL: coconut oil; EFA: linseed oil + safflower oil; CLA: Lutalin®; EFA + CLA: linseed oil + safflower oil + Lutalin®.

A total number of 260 oocytes were recovered after slicing the ovaries obtained from cows of each diet group. We did not supplement the *in vitro* culture media with any FA for IVP. As for IVP we purely analyzed the developmental competence of obtained oocytes *in vitro*. The percentage of oocytes which underwent cleavage 48 h after fertilization and the blastocyst rates at the 8th day are presented in Table 2. The cleavage rates of oocytes recovered from the EFA and CLA diet groups tended to be higher (51.7% ± 9.3% and 53.3% ± 5.1%) as compared to those of EFA + CLA (36.2% ± 10.6%) and of the CTRL (34 ± 8.1%), however without statistical significance. Similarly, the rate of blastocysts generated from the cleaved embryos in the CTRL (23.5% ± 11.7%) and in the EFA, CLA and EFA+CLA diet groups (17.4% ± 7.9%, 26.1 ± 11.2%, and 15.2% ± 8.4%, respectively) remained statistically indifferent. Blastocysts generated on day 8 post IVF from each diet groups were stained with Hoechst 33258 to assess the TCN (Figure 1). The TCN count in EFA, CLA and EFA + CLA were 92.1 ± 10.1, 124.9 ± 18.9, and 121 ± 13.2, respectively. as compared to TCN count of the CTRL (71.2 ± 12.1).

**Table 2:** Effects of dietary essential fatty acids on bovine *in vitro* embryo development.

**Diet group (No. of cows)**	**No. of oocytes (*n*)**	**(%) Cleavage rate (*n*)**	**(%) Blastocyst rate (*n*)**	**Total cell number (*n*)**
CTRL (7)	61	34 ± 8.1 (20)	23.5 ± 11.7 (6)	71.2 ± 12.1 (5)
EFA (7)	64	51.7 ± 9.3 (33)	17.4 ± 7.9 (7)	92.1 ± 10.1 (6)
CLA (8)	65	53.3 ± 5.1 (35)	26.1 ± 11.2 (12)	124.9 ± 18.9 (12)
EFA + CLA (8)	70	36.2 ± 10.6 (26)	15.2 ± 8.4 (7)	121 ± 13.2 (7)

*Values are means ± SEM. CTRL: coconut oil; EFA: linseed oil + safflower oil; CLA: Lutalin® EFA+CLA: linseed oil + safflower oil + Lutalin®*.

### Effect of ALA and *cis-9, trans-11* CLA on GC Functions *in vitro*

#### Steroid Hormone Production

**Figure 2:**
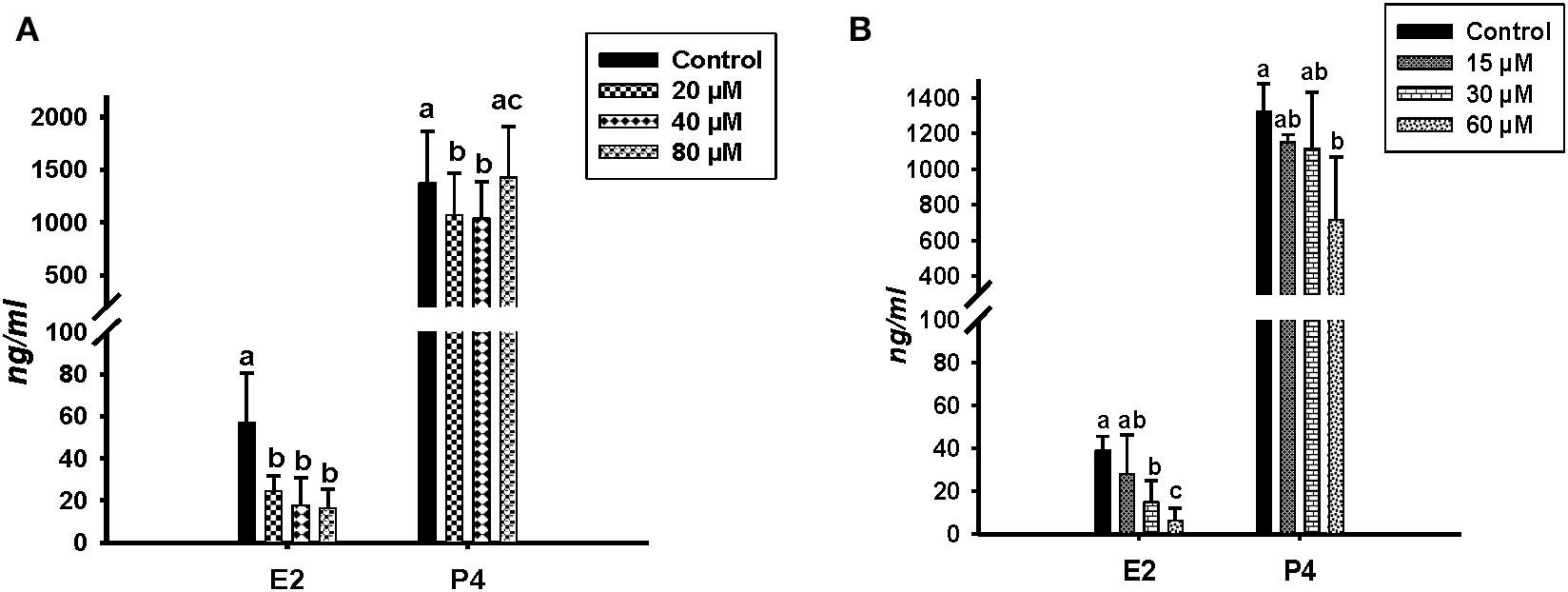
Effects of **(A)** ALA (20, 40, 80 μM) (*n* = 5) and **(B)** *cis-9, trans-11* CLA (15, 30, 60 μM) (*n* = 3) on 17β-Estradiol (E2) and progesterone (P4) concentrations (ng/ml) in conditioned media of cultured GC. Data are shown as means ± SEM; different lower case letters indicate significant differences between treatments *P* < *0.05*).

Concentrations of E2 and P4 were determined by RIA in the conditioned media of *in vitro* cultured GC treated with different concentrations of ALA and *cis-9, trans-11* CLA (Figure 2). ALA significantly reduced (*P* < *0.05*) the E2 production at all the concentrations as compared to the control (Figure 2A). *Cis-9, trans-11* CLA also reduced E2 significantly (*P* < *0.05*) at 30 and 60 μM as compared to the control (Figure 2B). P4 concentration was significantly reduced in GC treated with ALA at 20 and 40 μM (Figure 2A) whereas, P4 was only reduced (*P* < *0.05*) by *cis-9, trans-11* CLA at 60 μM (Figure 2B).

#### Cell Morphology and Gene Expression

**Figure 3:**
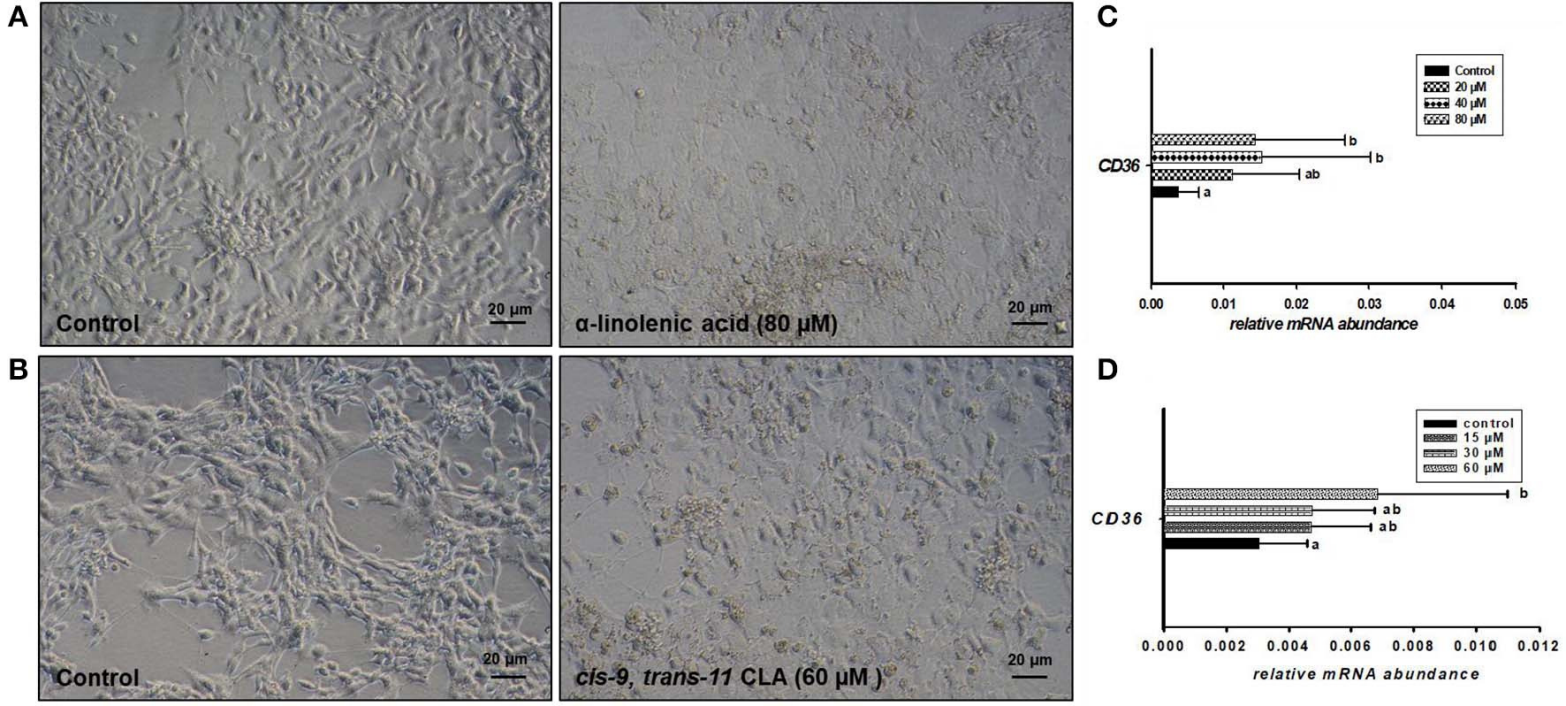
Effects of **(A)** ALA (80 μM) and **(B)** *cis-9, trans-11* CLA (60 μM) on the morphology of cultured GC. Photomicrographs were taken with a Nikon TMS-F inverted microscope. Effects of **(C)** ALA (20, 40, 80 μM) (*n* = 5) and **(D)** *cis-9, trans-11*CLA (15, 30, 60 μM) (*n* = 3) on *CD36* mRNA abundance. Gene expression was normalized to *RPLPO* transcripts. Data are shown as means ± SEM; different lower case letters indicate significant differences between treatments (*P* < *0.05*).

**Figure 4:**
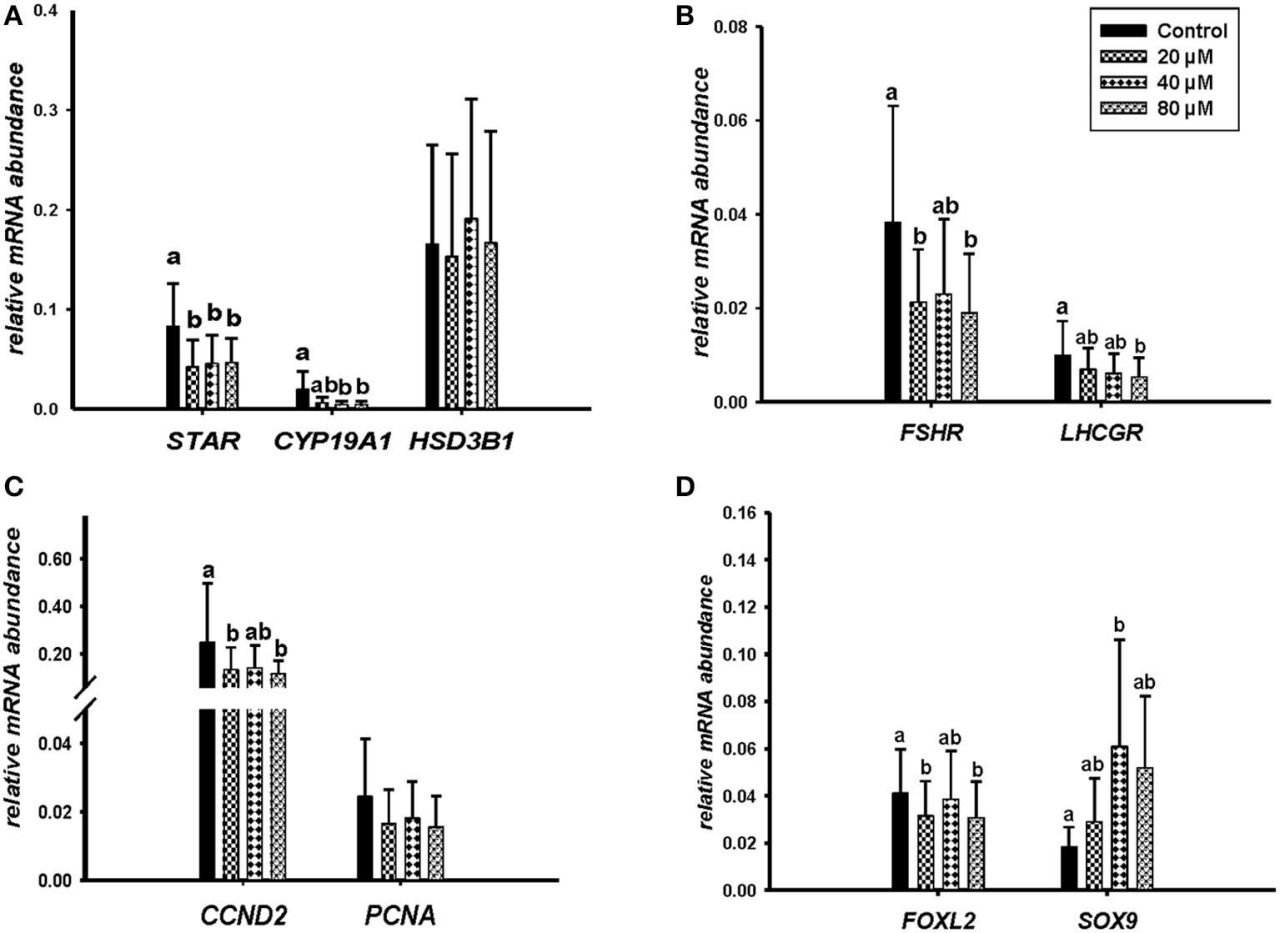
Effects of ALA (20, 40, 80 μM) on the expression of **(A)** key genes of steroidogenesis *STAR, CYP19A1, HSD3B1*, **(B)** gonadotrophin receptors *FSHR, LHCGR*, **(C)** cell proliferation *CCND2, PCNA*, and **(D)** on GC and sertoli cell markers *FOXL2, SOX9*. Gene expression was normalized to *RPLPO* transcripts. Data are shown as means ± SEM; different lower case letters indicate significant differences between treatments (*n* = 5, *P* < *0.05*).

**Figure 5:**
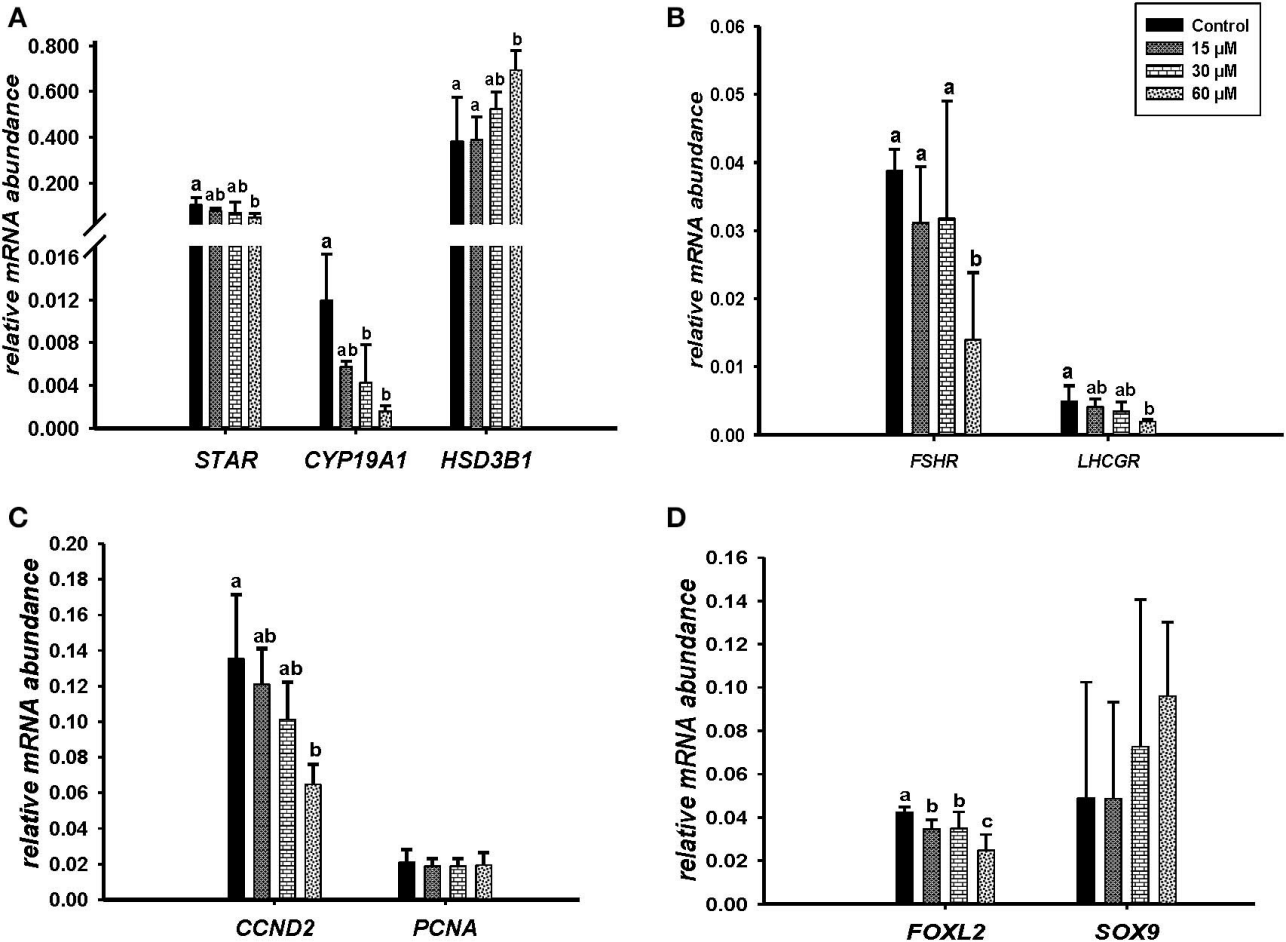
Effects of *cis-9, trans-11* CLA (15, 30, 60 μM) on the expression of **(A)** key genes of steroidogenesis *STAR, CYP19A1, HSD3B1*, **(B)** gonadotrophin receptors *FSHR, LHCGR*, **(C)** cell proliferation *CCND2, PCNA*, and **(D)** on GC and sertoli cell markers *FOXL2, SOX 9*. Gene expression was normalized to *RPLPO* transcripts. Data are shown as means ± SEM; different lower case letters indicate significant differences between treatments (*n* = 3, *P* < *0.05*).

Microscopic images taken on day 8 of *in vitro* culture showed that ALA and *cis-9, trans-11* CLA treatment induced intracellular lipid droplet accumulation in GC in particular at higher concentrations (Figures 3A,B). Further, RT-qPCR data revealed that transcript abundance of the fatty acid translocase *(CD36)* was strongly up-regulated by both ALA at 40, 80 μM (Figure 3C) and *cis-9, trans-11* CLA at 60μM (Figure 3D). ALA in GC significantly downregulated (*P* < 0.05) the key transcripts of estradiol production, steroidogenic acute regulatory protein (*STAR*) at all concentrations, cytochrome P450 Family 19 subfamily A member 1 (*CYP19A1*) at (40 and 80 μM), the follicle stimulating hormone receptor (*FSHR*) at 20 and 80 μM and the luteinizing hormone/choriogonadotropin receptor (*LHCGR*) at 80 μM (Figures 4A,B). While GC treated with cis-9, trans-11 CLA significantly downregulated (*P* < 0.05) *STAR* at 60 μM, *CYP19A1* at 30 and 60 μM, and both *FSHR* and *LHCGR* at 60 μM (Figures 5A,B). Hydroxy-delta-5-steroid dehydrogenase, 3 beta- and steroid delta-isomerase 1 *(HSD3B1)* gene expression remained unaltered in ALA (Figure 4A) treated GC while it was upregulated significantly in *cis-9, trans-11* CLA (60 μM) treated GC (Figure 5A). The mRNA expression of cyclin D2 (*CCND2)* was significantly downregulated by ALA in GC at 20 and 80 μM (Figure 4C), while *cis-9, trans-11* CLA downregulated *CCND2* at 60 μM (*P* < 0.05) as compared to the control (Figure 5C). Proliferating cell nuclear antigen (*PCNA)* mRNA expression remained unaltered at all tested concentrations in both ALA and *cis-9, trans-11* CLA treated GC (Figures 4C, 5C). The GC identity marker forkhead Box L2 (*FOXL2)* was significantly downregulated by ALA at 20 and 80 μM (Figure 4D) while *cis-9, trans-11* CLA downregulated the same at all concentrations as compare to control (Figure 5D). In contrast, SRY-Box 9 (*SOX9*) gene expression was upregulated significantly (Figure 4D) in ALA treated GC but seemed slightly affected in *cis-9, trans-11 CLA* treated GC (Figure 5D).

#### Cell Viability

**Figure 6:**
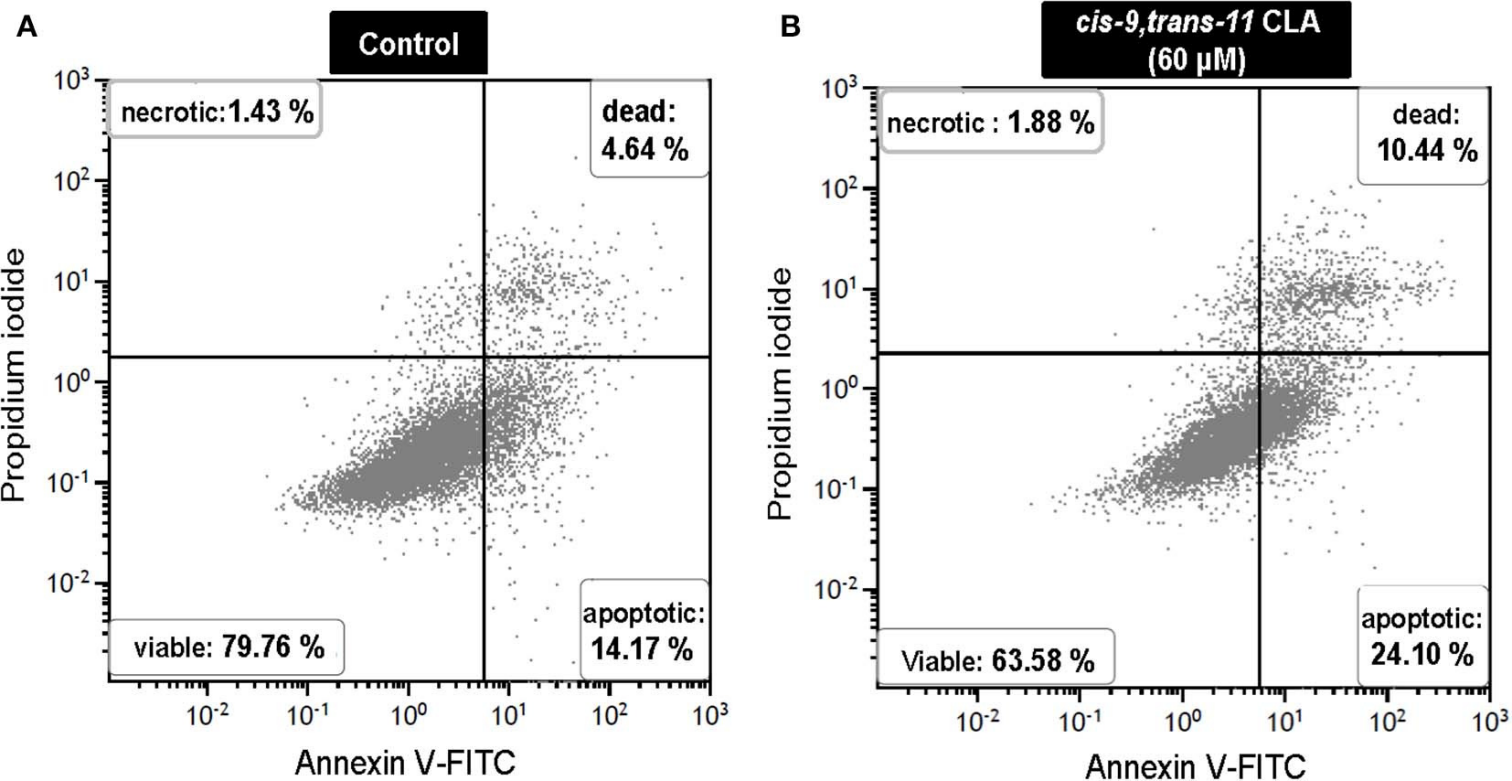
Representative dot plots of Annexin V and propidium iodide (PI) staining generated during a flow cytometry experiment. Annexin V and PI staining in **(A)** control and **(B)** *cis-9, trans-11* CLA (60 μM) cultured GC. Percentage of cells stained in each quadrant is listed in the corner of each quadrant.

Flow cytometry analysis of Annexin V-FITC/PI stained cells showed no significant change in percentage of viable, apoptotic or dead cells in GC treated with different concentrations of ALA as compared to the control. Whereas, GC treated with *cis-9, trans-11* CLA at 60 μM resulted in higher percentage of apoptotic cells (16.3 ± 3.1%) compared to control (10.5 ± 3%). Dot plots of Annexin V and propidium iodide (PI) staining generated during a typical flow cytometry experiment as shown in Figure 6. The percentage of dead cells in GC treated with *cis-9, trans-11* CLA at 30 and 60 μM (12.2 ± 2.2% and 12.4 ± 1.5%, respectively) were significantly higher as compared to the control (8 ± 1.6%). These results were well reflected by the percentage of viable cells going significantly down to 67.5 ± 2.8% in GC treated with 60 μM *cis-9, trans-11* CLA as compare to 78.3 ± 1.8% in the control (Table 3).

**Table 3:** Cell viability as determined by Annexin V-FITC/PI staining assay in bovine *in vitro* granulosa cells.

	**α-linolenic acid (ALA)**				***cis-9, trans 11*** **CLA**			
**Treatments**	**control**	**20 μM**	**40 μM**	**80 μM**	**control**	**15 μM**	**30 μM**	**60 μM**
Viable cells (%)	76.4 ± 0.5	70.4 ± 4.1	66.9 ± 2.6	73.7 ± 1.7	78.3 ± 1.8^**a**^	75.6 ± 3.9^**ab**^	71.7 ± 3.7^**ab**^	67.5 ± 2.8^**b**^
Apoptotic cells (%)	10.5 ± 3.5	10.8 ± 1.4	12.9 ± 5.4	10.6 ± 2.9	10.5 ± 3.0^**a**^	9.8 ± 2.5^**ab**^	11.9 ± 2.2^**ab**^	16.3 ± 3.1^**b**^
Dead cells (%)	9.4 ± 2.5	14.6 ± 4.4	14.1 ± 1.8	11.7 ± 1.0	8 ± 1.6^**a**^	11.2 ± 2.6^**ab**^	12.2 ± 2.2^**b**^	12.4 ± 1.5^**b**^

*Data analyzed by one-way repeated measure ANOVA, multiple comparison vs. control (n = 3; Dunnett's method). Values are means ± SEM. Different lower case letters on the same row indicate significant differences between treatments (P < 0.05)*.

## Discussion

Dietary lipid supplementation is supposed to improve fertility in dairy cows by increasing the size of the ovulatory follicle, plasma concentration of P4 and lifespan of CL (25) though, the influence on reproductive performance is by far not fully understood. In the present study, we analyzed the effects of dietary essential fatty acids on oocyte competence and GC function in bovine.

### Dietary Supplementation and Embryo Development

Following dietary supplementations, as expected we observed a higher percentage of ALA in the FF of EFA and EFA + CLA diet fed cows, while the percentage of the *cis-9, trans-11* CLA was higher in the FF of CLA and EFA+CLA diet fed cows as compared to the CTRL diet fed cows. It has been reported that cows when fed with encapsulated fats containing flaxseed oil had 5-fold higher concentrations of ALA in the FF and GC with increased numbers of follicles as compare to cows fed with encapsulated fats containing sunflower oil and control diet. Also, the cleavage rates in cows fed with flax seed oil were higher than in cows fed with a control diet (26). Similarly, cows fed with flaxseed oil contained higher amounts of ALA in FF, GC, and COCs and the percentage of oocytes that developed to blastocysts was also higher in flaxseed and fish oil fed cows as compared to cows that were fed saturated fats (27). In the present study we found higher proportions of ALA and *cis-9, trans-11* CLA in FF of EFA, CLA, and EFA + CLA diet group cows, however, these did not lead to significant improvements in the cleavage and blastocyst rates or TCN count. Our results revealed that neither EFA, CLA, or EFA + CLA diet supplements could improve the cleavage and blastocyst rates of *in vitro* generated embryos as compared to the CTRL diet supplement.

In previous studies, Holstein cows when fed with diets enriched in PUFA especially 18:2 and 18:3 FA despite improving dominant follicle size and CL volume failed to improve both cleavage and blastocyst rates of *in vitro* produced embryos (15). Similarly, feeding soybean oil (high in LA), or linseed oil (high in ALA) to lactating cows could not improve the blastocyst rate or TCN count of *in vitro* produced embryos compared to rumen inert fat (RIF) diet (28). Recent studies have provided new insights in effects of specific *in vitro* FA supplementation on oocyte maturation, cleavage and blastocyst rates. Supplementing *cis-*9, *trans-*11 or *trans-*10, *cis-*12 CLA isomer could not improve IVP, but embryos generated under *cis-*9, *trans-*11 CLA (100 μM) before vitrification were of improved quality (29). *In vitro* supplementation of ALA (25 μM) during IVM increased the porcine oocyte cleavage rate but at higher concentration of ALA (50 μM), though enhancing the nuclear maturation of oocytes it could not improve the cleavage, blastocysts rate or TCN. This suggests that high concentrations of ALA can adversely affect *in vitro* embryo development (30). In the present study we speculate that adverse effects of EFA and CLA diets on oocyte competence could be associated with higher proportions of ALA and *cis-9, trans-11* CLA as observed in the FF. Previous studies have documented the decisive role of FF components in determining oocyte quality and fertility in bovine and humans (31, 32). However, the small sample size could be a limitation in achieving decent blastocyst rates. Another plausible reason could be the experimental approach of oocyte retrieval from subordinate follicles. Since the ovaries were obtained from cows at different oestrous stage, which might affect their oocyte development competence as dominant follicle might suppress the subordinate follicles. And thus, the sliced follicles might not reflect the environment of the dominant preovulatory follicle. Furthermore, the selective dietary uptake of EFA and CLA supplementation leading to increased proportions of ALA and *cis-*9, *trans-*11 CLA in the follicular fluid might adversely affect developmental competence of oocytes residing within the follicles.

### ALA and *cis-9, trans-11* CLA Alter GC Morphology and the Expression of Identity Marker Genes

*In vitro* supplementation of ALA and *cis-9, trans-11* CLA induced significant alterations of the morphology of cultured GC. The morphology of the cells was similar to oleid acid (OA) treated GC that we already reported earlier (33). These morphological changes are in line with the observation of an increased mRNA abundance of fatty acid translocase, *CD36*, which is known to mediate fatty acid uptake (34). The observed results are also consistent with reports in other mammalian cell lines. *Cis-9, trans-11* CLA induced lipid accumulation in human macrophages and in mice 15P-1 cell lines (testicular cells) along with an elevated expression of *CD36* (35, 36). ALA enriched diets increased the transport of lipids into resting skeletal muscles in conjunction with increased sarcolemmal ω-3 PUFA content and *CD36* protein expression in rats (37). This suggests that both ALA and *cis-9, trans-11* CLA are well-accumulated in mammalian cells either *in vitro* or *in vivo* as diet supplements.

The transcriptional regulators, *FOXL2* and *SOX9* are well-associated to each other (38) as *FOXL2* prevents transdifferentiation of the adult ovary to testis. Inducible deletion of *FOXL2* in adult ovarian follicles can upregulate the testis-specific marker gene *SOX9* (39). Previously, we reported that OA markedly reduced the transcription of *FOXL2* and increased *SOX9* in cultured GC (40). A similar regulation was observed in the present study with ALA *and cis-9, trans-11 CLA* supplementation decreasing the mRNA abundance of *FOXL2* and upregulating *SOX9* transcripts. Apart from being an GC identity marker *FOXL2* is also known to regulate ovarian steroid metabolism (41). The presented results clearly indicate that both, ALA and *cis-*9, *trans-11* CLA modulate the *FOXL2* transcriptional activity. Thus, the role of *FOXL2* in regulating the lipid metabolism in terms of promoting lipid droplet formation in GC upon ALA *and cis-9, trans-11* CLA treatment could be well-anticipated from the presented data. The results suggest that both ALA and *cis-9, trans-11 CLA* impose alike effects on the transcript levels of *FOXL2* in GC which might further affect other downstream target molecules involved in normal GC function.

### ALA and *cis-9, trans-11* CLA Modulate GC Steroidogenesis

For normal GC function, steroidogenesis is initiated by the transportation of free cytoplasmic cholesterol into mitochondria by the protein *STAR* (42). Successive steps are then catalyzed by cholesterol side chain cleavage enzyme encoded by cytochrome P450 side-chain cleavage enzyme (*CYP11A1)*, by 3 beta hydroxyl steroid dehydrogenase transcribed from *HSD3B1*, and by *CYP19A1* encoding the key enzyme of E2 synthesis (43). Steroidogenesis is a vital part of folliculogenesis as studied in knockout mouse models (44). Also FSHR and LHCGR play a vital role during follicle maturation (45).

In the present study, we determined the effects of ALA and *cis-9, trans-11* CLA on steroid hormone production, on transcriptional activity of key genes involved in steroidogenesis (*CYP19A1, STAR*, and *HSD3B1)* and gonadotropin hormone signaling (*FSHR* and *LHCGR)* in cultured GC. The transcript level of *STAR* as well as the E2 production was significantly downregulated by both ALA and *cis-9, trans-11* CLA. Most likely, the reduction in E2 concentrations can be attributed to reduced transcription of *CYP19A1* as observed after ALA and *cis-9, trans-11* CLA treatment. CLA has been shown to be very potent in reducing *CYP19A1* expression and E2 production in buffalo GC as well (46). Another PUFA, arachidonic acid (AA) at 50 μM stimulates the proliferation of bovine GC by activating both extracellular signal-regulated kinases 1/2 (ERK1/2) and Akt signaling pathway, however, higher dose decreases E2 secretion and downregulates the mRNA abundance of *CYP19A1, FSHR, HSD3B1*, and *STAR* in cultured bovine GC (47). This, suggest that PUFA at higher levels might modulate steroidogenesis by intracellular signaling pathways that regulate target gene expression. FSH is known to induce the expression of *LHCGR* via protein kinase A (PKA) and phosphoinositide3-kinase (PI3K) pathways in rat GC (48). *LHCGR* is highly expressed in GC during the pre-ovulatory stage to enable responsiveness to the LH surge thus leading to ovulation, oocyte maturation and CL formation (49). The down-regulation of *FSHR* and *LHCGR* by both FA at higher concentrations might affect follicle stimulating hormone (FSH) signaling and steroidogenesis in GC as suggested by our results. *CYP11A1* converts cholesterol into pregnenolone and *HSD3B1* converts pregnenolone into P4 (50). *HSD3B1* gene expression though remained unaltered in GC treated with ALA but P4 production was reduced. Similar effects on P4 production have been observed in primary goat GC treated with ALA at 100 μM (51). Surprisingly, *cis-9, trans-11* CLA significantly upregulated *HSD3B1* gene expression while reducing the P4 concentration in conditioned media. This outcome might be due to negative feedback effects of P4 production on *HSD3B1* transcription. Together, these results suggest that high levels of ALA or *cis-9, trans-11* CLA in FF can adversely affect GC by reducing both E2 and P4 production, which in turn could result in compromised ovarian cyclicity and impaired fertility in lactating cows. This is also in line with our recent study, where we could show that increased concentrations of OA in the follicular fluid can actually impair the functionality of GC *in situ*, thus obviously suppressing ovulation (21).

### *cis-9, trans-11* CLA Induces Apoptosis in GC

It is well-documented that higher E2 levels are capable of protecting GC from Fas ligand induced apoptosis and promote the cell transit from G1 to S phase with increased *CCND2* expression (52, 53). In our results, GC morphology was altered upon ALA and *cis-9, trans-11* CLA treatment, and both ALA and *cis-9, trans-11*CLA could down-regulate *CCND2* mRNA expression. However, only *cis-9, trans-11*CLA could elicit significant apoptotic effects in GC. As reported earlier, CLA is known to induce apoptosis in human, mouse and rat cell lines (54–57). *CCND2* mRNA expression is FSH dependent as reported in FSHR null mutant mice exhibiting decreased *CCND2* mRNA levels (58). This goes well in line with our observation that *cis-9, trans-11* CLA treated cells not only reduced E2 production, but also reduced *FSHR* mRNA expression followed by a steep downregulation of *CCND2* mRNA expression.

In contrast, ALA despite of reducing the E2 levels and *CCND2* mRNA abundance did not induce any apoptotic effects in GC. This observation suggests the importance of FSH signaling during cell cycle regulation of GC. However, proliferating cell nuclear antigen (PCNA) gene expression remained unaltered in GC treated with both ALA and *cis-9, trans-11* CLA. According to previous immunocytochemistry studies different *cis-9,trans-11* CLA concentrations at different time periods could significantly decrease the *PCNA* expression and inhibit cell growth and proliferation in mammary cancer cells (MCF-7 cells) (59) and in gastric adenocarcinoma cell lines (SGC-7901) (60). However, these effects were only observed on translation/protein levels as changes of transcript abundance were not determined.

## Conclusion

We investigated the effects of dietary essential fatty acids on bovine oocyte competence and GC functionality. Essential fatty acid supplementation could not improve the embryo IVP in spite of a substantial increase of ALA and *cis-9, trans-11* CLA in the follicular fluid. Further, the study clearly suggests that GC morphology and functionality could be considerably affected with increased follicular proportions of ALA and *cis9, trans-11*CLA particularly constraining steroidogenesis in GC. *In vivo* this may result in a compromised ovarian cyclicity and impaired fertility.

## Data Availability Statement

All datasets generated for this study are included in the article/Supplementary Material.

## Ethics Statement

The animal study was reviewed and approved by federal state of Mecklenburg Western-Pommerania, Germany LALLF M-V TSD 7221.3-1-038/15.

## Author Contributions

HH designed the dietary supplements for the cows. AV and VR performed the ovarian follicular fluid collection. DD and TV executed the fatty acid analysis. JS and AS executed the IVF experiments. AS, VB, and JV designed and executed the *in vitro* cell culture experiments. AS and JV primarily wrote the manuscript, with all co-authors making significant contribution to the final manuscript.

### Conflict of Interest

The authors declare that the research was conducted in the absence of any commercial or financial relationships that could be construed as a potential conflict of interest.
