# Supplementary material for: Effects of Dietary Fatty Acids on Bovine Oocyte Competence and Granulosa Cells
Source: Front Endocrinol (Lausanne). 2020 Feb 25;11:87. doi: 10.3389/fendo.2020.00087 (PMC7052110; doi:10.3389/fendo.2020.00087)
Supplement: Supplementary file 1 [file Table_1.DOCX]

**Supplementary Table 1.** Ingredients and chemical composition of lactation and dry-off diet

|  | **Diet** | |
| --- | --- | --- |
| **Item (g/kg of DM)** | **Lactation** | **Dry-off^1^** |
| **Ingredients** |  |  |
| Corn silage | 457 | 421 |
| Straw | 97 | 223 |
| Compound feed DEFA^2^ (granulated) | 446 | - |
| Dried sugar beet pulp | - | 163 |
| Extracted soy bean meal | - | 99 |
| Grain of rye | - | 75 |
| Minerals^3^ | - | 10 |
| Urea^4^ | - | 9 |
| Chemical composition^5^ |  |  |
| NEL (MJ/kg DM) | 7.1 | 6.5 |
| Crude fat | 23 | 21 |
| Crude fiber | 173 | 219 |
| Crude protein | 146 | 141 |
| Utilizable protein | 143 | 141 |
| NDF | 346 | 423 |
| ADF | 197 | 249 |

^1^The Dry period diet was fed from wk 6 to 0 before calving

^2^Ceravis AG, Malchin, Germany: 46.5% dried sugar beet pulp, 25.3% extracted soy bean meal, 23.8% grain of rye, 1.4% urea, 1.1% premix cow, 1.00% calcium, 0.37% phosphorus, 0.42% sodium, vitamins A, D3, E, copper, ferric, zinc, manganese, cobalt, iodine, selenium; 9.3% crude fiber, 8.2% crude ash, 1.8% crude fat, 24.1% crude protein, 21.6% NDF, 12.4% ADF, 7.9 MJ NE_L_/kg DM

^3^KULMIN^®^MFV Plus (Bergophor Futtermittelfabrik Dr. Berger GmbH & Co. KG, Kulmbach, Germany): 8.5% magnesium, 7.5% phosphorus, 6.5% sodium, 3.5% HCl-insoluble ash, 1.5% calcium, additives: vitamins A, D_3_, E, B_1_, B_2_, B_6_, B_5_, B_3_, B_12_, B_9_, H, zinc, manganese, copper, cobalt, iodine, selenium, saccharomyces cerevisiae

^4^Piarumin^®^ (SKW Stickstoffwerke Piesteritz GmbH, Lutherstadt Wittenberg, Germany): 99 % urea, 46.5 % total nitrogen

^5^German Society of Nutrition Physiology (2001)
