# Supplementary material for: Effects of Dietary Fatty Acids on Bovine Oocyte Competence and Granulosa Cells
Source: Front Endocrinol (Lausanne). 2020 Feb 25;11:87. doi: 10.3389/fendo.2020.00087 (PMC7052110; doi:10.3389/fendo.2020.00087)
Supplement: Supplementary file 2 [file Table_2.DOCX]

**Supplementary Table 2:** Fatty acid composition of the supplemented lipids

| **Fatty acid (%)** | **Coconut** | **Linseed** | **Safflower** | **Lutalin®** |
| --- | --- | --- | --- | --- |
| SFA | 89.4 | 10.4 | 12.3 | 11.1 |
| 6:0 | 0.93 | − | − | − |
| 8:0 | 9.85 | − | − | − |
| 10:0 | 6.10 | − | − | − |
| 12:0 | 45.5 | − | − | − |
| 14:0 | 16.9 | 0.02 | 0.09 | − |
| 16:0 | 6.87 | 5.78 | 7.63 | 6.23 |
| 17:0 | − | 0.03 | − | − |
| 18:0 | 3.10 | 4.11 | 3.71 | 4.19 |
| 20:0 | 0.11 | 0.19 | 0.42 | 0.22 |
| 22:0 | − | 0.13 | 0.25 | 0.37 |
| 24:0 | − | 0.09 | 0.16 | 0.10 |
| MUFA | 8.35 | 21.5 | 23.5 | 29.5 |
| 16:1, *cis*-9 | − | 0.07 | 0.12 | 0.09 |
| 18:1, *cis*-9 | 8.35 | 20.7 | 22.4 | 28.3 |
| 18:1, *cis*-11 | − | 0.61 | 0.66 | 0.67 |
| 20:1, *cis*-11 | − | 0.16 | 0.23 | 0.51 |
| 24:1, *cis*-15 | − | − | 0.11 | − |
| PUFA | 1.83 | 67.1 | 62.5 | 3.75 |
| 18:2, *cis*-9, *cis*-12 | 1.83 | 15.9 | 62.0 | 3.52 |
| 18:2, *cis*-9, *trans*-12 | − | − | − | 0.08 |
| 18:2, *trans*-9, *cis*-12 | − | − | − | 0.07 |
| 18:3, *cis*-6, *cis*-9, *cis*-12 | − | − | 0.05 | 0.07 |
| 18:3, *cis*-9, *cis*-12, *cis*-15 | − | 51.1 | 0.24 | − |
| 20:2, *cis*-11, *cis*-14 | − | 0.06 | − | − |
| 20:3, *cis*-11, *cis*-14, *cis*-17 | − | 0.02 | − | − |
| 22:5, *cis*-7, *cis*-10, *cis*-13, *cis*-16, *cis*-19 | − | − | 0.28 | − |
| CLA | < 0.05 | 0.02 | 0.32 | 54.3 |
| 18:2, *cis9, trans11* CLA | − | − | 0.12 | 27.2 |
| 18:2, *trans10, cis12* CLA | − | 0.02 | 0.20 | 27.0 |
| 18:2, *cis9, cis11* CLA | − | − | − | 0.23 |

SFA: saturated fatty acid; MUFA: monounsaturated fatty acid; PUFA: polyunsaturated fatty acid; CLA: conjugated linoleic acid Control (CTRL, n=9): 76 g/d coconut oil (Bio-Kokosöl #665, Kräuterhaus Sanct Bernhard KG, Bad Ditzenbach, Germany) and 0.06 g/d Vitamin E (Covitol®1360, BASF SE, Ludwigshafen, Germany), 1.48 MJ NE_L_/d

Essential fatty acids (EFA, n = 9): 78 g/d linseed (DERBY® Leinöl #4026921003087, DERBY Spezialfutter GmbH, Münster, Germany) and 4 g/d safflower oil (GEFRO Distelöl, GEFRO Reformversand Frommlet KG, Memmingen, Germany), comprised 0.06 g/d Vitamin E, 1.57 MJ NE_L_/d

Conjugated linoleic acid (CLA, n = 10): 38g/d Lutalin^®^ (BASF SE, Ludwigshafen, Germany) and 0.06 g/d Vitamin E (Covitol®1360, BASF SE, Ludwigshafen, Germany), 0.69 MJ NE_L_/d

Essential fatty acids and conjugated linoleic acid (EFA+CLA, n = 10): 78 g/d linseed (DERBY® Leinöl #4026921003087, DERBY Spezialfutter GmbH, Münster, Germany),4 g/d safflower oil (GEFRO Distelöl, GEFRO Reformversand Frommlet KG, Memmingen, Germany) and 38 g/d Lutalin® (BASF SE, Ludwigshafen, Germany), comprised 0.06 g/d Vitamin E, 2.26 MJ NE_L_/d
