# Supplementary material for: Effects of Dietary Fatty Acids on Bovine Oocyte Competence and Granulosa Cells
Source: Front Endocrinol (Lausanne). 2020 Feb 25;11:87. doi: 10.3389/fendo.2020.00087 (PMC7052110; doi:10.3389/fendo.2020.00087)
Supplement: Supplementary file 3 [file Table_3.DOCX]

**Supplementary Table 3:** List of Primers used for transcript quantification by RT-qPCR

| **Gene** | **Sequence** | **Size (bp)** | **Accession No.** |
| --- | --- | --- | --- |
| *RPLPO* | For: TGGTTACCCAACCGTCGCATCTGTA  Rev: CACAAAGGCAGATGGATCAGCCAAG | 142 | NM_001012682 |
| *STAR* | For: TTGTGAGCGTACGCTGTACCAAG  Rev: CTGCGAGAGGACCTGGTTGATG | 237 | NM_174189.2 |
| *CYP19A1* | For: GCTTTTGGAAGTGCTGAACCCAAGG  Rev: GGGCCCAATTCCCAGAAAGTAGCTG | 172 | NM_174305 |
| *HSD3B1* | For: TGTTGGTGGAGGAGAAGGATCTG Rev: GCATTCCTGACGTCAATGACAGAG | 208 | NM_174343 |
| *FSHR* | For: TCACCAAGCTTCGAGTCATCCCAAA Rev: TCTGGAAGGCATCAGGGTCGATGTA | 189 | NM_174061 |
| *LHCGR* | For: GCATCCACAAGCTTCCAGATGTTACGA Rev: GGGAAATCAGCGTTGTCCCATTGA | 205 | NM_174381 |
| *CD36* | For: GCTCCTTAAGCCATTCTTGGAT Rev: CACCAGTGTCAACGCACTTT | 151 | NM_001278621.1 |
| *CCND2* | For: CGCAGGGCCGTGCCGGACGCCAAC Rev: CACGGCCCCCAGCAGCTGCAGATGG | 279 | NM_001076372 |
| *PCNA* | For: GTGAACCTGCAGAGCATGGACTCGT  Rev: CGTGTCCGCGTTATCTTCAGCTCTT | 192 | NM_001034494 |
| *FOXL2* | For: AGCCAAGTTCCCGTTCTACG  Rev: GGTCCAGCGTCCAGTAGTTG | 140 | NM_001031750.1 |
| *SOX 9* | For: ACCTGGAACTTCAGTGGCG  Rev: CCAAGTAGGGGAAGGCGAAT | 147 | XM_010816647.1 |
